# En bloc Extended Total Thymectomy and Extrapleural Pneumonectomy in Masaoka stage IVA Thymomas

**DOI:** 10.1186/1749-8090-6-28

**Published:** 2011-03-12

**Authors:** Hee Chul Yang, Yoo Sang Yoon, Hong Kwan Kim, Yong Soo Choi, Kwhanmien Kim, Young Mog Shim, Jungho Han, Jhingook Kim

**Affiliations:** 1 Department of Thoracic and Cardiovascular Surgery, Samsung Medical Center, Sungkyunkwan University School of Medicine, Seoul, South Korea; 2 Department of Thoracic and Cardiovascular Surgery, Seoul National University Bundang Hospital, Seoul National University College of Medicine, Gyeonggi-do, South Korea; 3 Department of Pathology, Samsung Medical Center, Sungkyunkwan University School of Medicine, Seoul, South Korea

## Abstract

**Background:**

Surgical excision is the primary treatment for a thymoma. However, for advanced thymoma that extends to within the thoracic cavity and for recurrent cases with pleural dissemination (Masaoka stage IVA), the appropriate treatment is controversial. We evaluated the safety of surgery and outcomes of seven patients that underwent an en bloc extended total thymectomy and extrapleural pneumonectomy for stage IVA thymomas.

**Methods:**

From 1994 to 2009, five patients initially diagnosed with pleural dissemination and two patients with recurrent tumors in the pleura and lungs after a total thymectomy, were identified. Seven patients had an extrapleural pneumonectomy performed. For the first operation, five patients underwent additional en bloc extended total thymectomy.

**Results:**

Two recurrent cases were identified 55.2 and 12.3 months after first operation. Two patients had WHO type B1-B2 tumors, two had B2, two had B2-B3, and one had a B3 tumor. The mean hospital stay was 15.3 days (range: 7-29). There was no operative mortality. Four patients had neoadjuvant chemotherapy and five were treated with adjuvant chemotherapy. The median survival was 30.6 months and the Kaplan-Meier 2-year survival was 100% (95% confidence interval: 24.6-36.6 months). One patient, who did not receive induction chemotherapy, had distant metastases after surgery.

**Conclusions:**

En bloc extended total thymectomy and extrapleural pneumonectomy can be safely performed on selected patients with stage IVA thymomas and is expected to achieve complete local control. Although the treatment strategy has yet to be standardized, complete resection with appropriate systemic therapy may improve survival in otherwise fatal disease.

## Background

The prognosis of locally advanced thymomas within the thoracic cavity (Masaoka stage IVA) is poor [1-3]; there is no reliable treatment strategy established to date [4,5]. All three common therapeutic modalities (surgery, chemotherapy and radiation) can be used for the treatment of locally advanced thymoma [6,7]. However, the combination of these modalities has not been standardized.

Complete local control is the mainstay of treatment for a thymoma; this is because thymomas rarely metastasize to distant organs. For stage IVA thymomas, the tumor has not yet spread to extrathoracic organs and is still locally advanced. This stage allows for complete eradication. However, in miliary or confluent pleural disseminated thymomas, complete resection is almost impossible with a simple pleurectomy. In these cases, only extrapleural pneumonectomy (EPP) can resect all gross implants. EPP has also been performed in other malignant pleural tumors such as mesotheliomas. The aim of this retrospective study was to evaluate the safety and the long term efficacy of en bloc extended total thymectomy and EPP procedures for stage IVA thymomas.

## Methods

A retrospective review of all patients undergoing resection for thymic epithelial tumors, at a single center between January 1994 and December 2009, was performed. During this time period, 335 patients underwent surgery for the treatment of a thymoma. There were 19 patients (5.7%) diagnosed with stage IVA thymomas. Simple parietal pleurectomy and resection of the involved lung were performed in seven patients, debulking surgery in two, and biopsy only in three. Seven consecutive patients (2.1%) underwent EPP for Masaoka stage IVA thymomas and were included in this analysis. The patient characteristics are listed in Table 1.

**Table 1:** Patient Characteristics, Treatment, and Outcome

Patient (age, sex)	Side/Year/Histology	Previous treatment	Resection status	Adjuvant treatment	Recurrence site	Outcome, follow-up
1 (34, F)	Rt/2005/B1+B2	None	Complete	None	Peritoneum 13 mo	DOD, 25 mo
2 (58, M)	Rt/2005/B2	S/adjuvant RT (54Gy)	Complete	None	None	Dead, pneumonia, 27 mo
3 (35, M)	Rt/2006/B2+B3	S (sternotomy, stage III)	Complete	CT (CAP)	None	NED, 40 mo
4 (65, F)	Lt/2007/B2	CT (CAV #2, VIP #10)	Incomplete	CT+RT (60Gy)	Mediastinum, pleura	DOD, 31 mo
5 (49, F)	Lt/2007/B1+B2	CT (docetaxel, cisplatin)	Complete	CT (CAP)	None	NED, 27 mo
6 (50, M)	Rt/2007/B2+B3	CT (docetaxel, cisplatin)	Complete	CT (CAP)	None	NED, 24 mo
7 (52, M)	Lt/2008/B3	CT (docetaxel, cisplatin)	Complete	CT (CAP)	None	NED, 13 mo

DOD, dead of disease; NED, no evidence of disease;

S, surgery; RT, radiotherapy; CT, chemotherapy;

CAP, cyclophosphamide, doxorubicin, cisplatin; CAV, cyclophosphamide, doxorubicin, vincristine;

VIP, etoposide, ifosfamide, cisplatin.

Among the seven patients, patient 2 and 3 were treated for a recurrent thymoma in the pleural cavity. The other five patients that initially presented with a mediastinal thymoma and pleural dissemination, underwent an en bloc extended total thymectomy and EPP.

None of the patients had extrathoracic metastatic disease by the whole body positron emission tomography - computed tomography (PET-CT). All patients were thought to be able to tolerate the pneumonectomy in terms of heart and lung function. There were no other significant medical problems such as myasthenia gravis. EPP was performed by thoracotomy; pericardial reconstruction with a bovine pericardium and reconstruction of the diaphragm with a polytetrafluoroethylene (PTFE) patch was carried out. Patients 1, 4 and 6 underwent an en bloc extended total thymectomy and EPP by thoracotomy only. In two cases (patient 5 and 7) with invasion of the innominate vein, a median sternotomy was added for en bloc extended total thymectomy immediately after resecting the lungs and pleura via a posterolateral thoracotomy. Complete resection was defined as resection of all gross tumors with negative margins on pathology. Our institutional review board granted approval for this study on April 22, 2010.

A follow up computed tomography (CT) of the chest was obtained at three and six months after surgery and then a PET-CT was performed at 12 months. One year after the operation, a chest CT was performed every 6 months and PET-CT every 12-18 months in the absence of changes in the clinical condition. The date of recurrence was determined from the first postoperative radiological images that showed evidence of recurrence. Survival was calculated from the date of the operation to the date of death or to the date of the last follow up and was estimated by the Kaplan-Meier method using SPSS 17.0 (SPSS Inc, Chicago, Ill) software.

## Results

The median patient age was 50 (range: 34-65 years). Four patients were male. There were right pleural lesions in four cases. Mean operative time was 431 ± 61 minutes (range: 372-533 minutes) and bleeding loss during the procedure was mean 1210 ± 561 cc. There was no operative mortality and no additional morbidity except for one patient with postoperative delirium. All patients were discharged from the hospital without any significant problems. The mean postoperative hospital stay was 15 days (range: 7-29 days).

According to the pathology report, two patients had WHO type B1-B2 tumors, two had B2, two had B2-B3, and one had a B3 tumor. The mean of the largest mass was 9.9 cm (range: 6-13 cm). None of the patients had lymph node metastasis. Complete resection was achieved in six (85.7%) patients.

### Patient Descriptions

#### Patient 1

A 38-year-old woman presented with the superior vena cava (SVC) syndrome. About an 11 cm sized thymoma with right pleural dissemination invaded the SVC. Without induction chemotherapy, en bloc extended total thymectomy and a right EPP with SVC reconstruction was performed via a posterolateral thoracotomy. This patient had no neoadjuvant chemotherapy because she was a young patient and especially had manifested symptomatic SVC syndrome. In addition, it was difficult to predict the effect of neoadjuvant chemotherapy, therefore we performed surgery first and decided to discuss about the necessity of adjuvant chemotherapy. The patient had a satisfactory recovery. However, recurrence was found in the abdominal cavity 13 months after the en bloc resection. The patient refused systemic therapy and died of disease progression 25 months after surgery.

#### Patient 2

A 62-year-old man underwent a video assisted thoracoscopic (VATS) thymectomy (Masaoka stage II, WHO B2, mass size 5.3 cm) followed by adjuvant radiotherapy (54Gy). Fifty five months post surgery, pleural recurrence developed and a right EPP was performed. The patient recovered uneventfully. However, he died due to pneumonia caused by cerebral infarction 27 months post surgery.

#### Patient 3

A 39-year-old man underwent thymectomy via a median sternotomy (Masaoka stage III, WHO B2+B3) at another hospital. One year after the operation, the patient was referred to this hospital because he had pleural seeding with invasion of the right atrial wall. A right EPP with partial resection and primary closure of the right atrial wall was performed and then followed by adjuvant chemotherapy (4 cycles). The patient was alive without recurrence at 40 months after the EPP.

#### Patient 4

A 68-year-old woman who was diagnosed with a stage IVA thymoma received chemotherapy (12 cycles) at a different hospital. The patient was referred to this hospital for a chemoresistant tumor. An en bloc extended total thymectomy and Left EPP were performed. Residual tumor around the innominate vein was left in place because of tight adherence. The patient received adjuvant chemoradiotherapy. However, she died of disease progression 31 months after surgery.

#### Patient 5, 6 and 7

The most recent three patients that had mediastinal thymomas with pleural dissemination at initial presentation were enrolled and received a standardized multidisciplinary approach to treatment. All patients had induction chemotherapy (3 cycles) followed by en bloc extended total thymectomy with EPP and then adjuvant chemotherapy (3 or 4 cycles). All of these patients are still alive without any recurrences.

**Figure 1:**
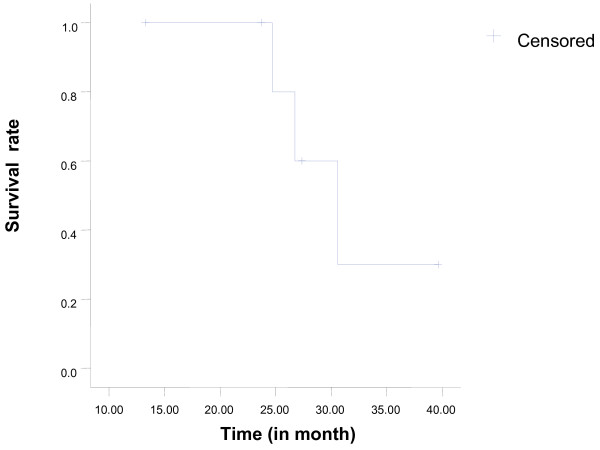
**Overall survival in the 7 patients that underwent EPP for stage IVA thymoma**.

The median survival was 30.6 months and the Kaplan-Meier 2-year survival was 100% (95% confidence interval: 24.6-36.6 months). However, among the three patients that were followed for over three years, one patient died of distant metastasis in the abdominal cavity, another patient died of pneumonia, and the other is alive without disease recurrence. The survival curve is shown in Figure 1.

## Discussion

An 11 year (1995-2005) experience with thymic epithelial tumors was previously reported [8]. The 5-year survival rate for a Masaoka stage IV (including IVA and IVB) thymoma was only 52%, which is significantly lower compared to stages I (96%), II (100%) and III (71%) tumors. The complete resection rate was also low (26.3%) for stage IV thymomas compared to stages I (100%), II (100%) and III (85.3%) tumors. The reason for the poor prognosis of stage IV thymomas was incomplete resections. Despite the fact that complete resection has been identified as a prognostic factor associated with long term survival of patients [9,10], few studies have been carried out in patients with stage IVA thymomas due to the difficulty of achieving a total resection, its rare occurrence and its indolent natural history.

Stage IVA patients can be treated with primary chemotherapy, radiotherapy [11] or chemoradiotherapy [12] without surgical resection, as well as debulking surgery with adjuvant radiation [13]. Although several investigators have attempted to improve the survival of patients with locally advanced thymoma, the data has been inconsistent with regard to the response rate and survival. Our approach is with aggressive surgery to eradicate the tumor. The problem lies in how a thymoma with extensive pleural dissemination, should be treated; in these cases, complete resection is not possible by simple parietal pleurectomy and lung preserving surgery. The most reliable resection method for stage IVA thymomas might be the EPP; because this procedure can remove invisible tumor cells as well as all gross implants.

Recent studies have reported favorable experiences with the EPP for cure of stage IVA thymomas [14-16]. The EPP for stage IVA thymomas has been performed at this hospital since 2005. This series had no cardiorespiratory morbidities, no perioperative death, and a reasonable hospital stay. These favorable results may be due to relatively young patients, with a good performance status and no underlying pulmonary disease in addition to the meticulous postoperative care.

Wright and colleagues [14] carried out EPP in five stage IVA patients. The five-year survival rate was reported to be 75% and was 50% for 10-years, which was fairly good compared to previous reports. Ishikawa and colleagues [16] reported 11 patients with invasive thymomas disseminated into the pleural cavity that underwent multimodality therapy. The patients that underwent EPP (n = 4) had better local recurrence free survival compared to the patients that did not have an EPP (n = 7) (5-year: 75% vs. 16%, 10-year: 75% vs. 0%). Huang and colleagues [15] reported on multimodality therapy in 18 patients with stage IVA thymomas. Complete resections were performed in 12 patients including nine pleurectomies and three EPP procedures. Among three out of the nine patients with pleurectomies, recurrences developed in the pleura. By contrast, three patients that underwent an EPP were alive without disease recurrence at 4, 32, and 112 months. These results suggest that the EPP achieves a higher complete local control rate than other surgical procedures. Among the completely resected patients in this series, distant metastases developed in one patient, who was the first EPP case at this hospital, and had no systemic therapy. After this experience, systemic therapy for stage IVA thymomas became an important part of treatment.

Many studies suggest a multimodality approach may lower the recurrence rate and increase the resectability of advanced thymomas and this approach is currently widely accepted [6,7,15,17]. However, how to combine these modalities remains controversial in patients with stage IVA thymomas. We concluded that an optimal treatment sequence for stage IVA thymomas might include induction chemotherapy, en bloc extended total thymectomy with EPP, and adjuvant chemotherapy. The three most recent consecutive patients (patients 5, 6 and 7) were treated by the protocol discussed above. They received the same chemotherapy agents (doxetaxel and cisplatin) for induction chemotherapy and they all had a partial response. Complete resection was performed by an en bloc extended total thymectomy and EPP. Adjuvant chemotherapy included CAP (cyclophosphamide, doxorubicin and cisplatin), and all the patients are currently alive without disease recurrence. The patients that were planned to have EPP did not receive preoperative radiotherapy. Preoperative radiotherapy may have adverse effects on the postoperative outcomes because it can damage the heart and lungs with the wide extent of the radiation field needed for treatment. Radiotherapy was performed in only one patient with macroscopic residual tumor around the innominate vein. Hemithoracic radiation was not carried out in this series. All of the patients that received adjuvant chemotherapy alone (n = 4) had no recurrence. Huang and colleagues [15] treated four patients with induction chemotherapy followed by EPP and then adjuvant hemithoracic radiation for stage IVA thymomas. Wright [14] suggested induction chemotherapy followed by EPP, and then adjuvant chemoradiation. However, further study of the role of adjuvant chemotherapy or radiotherapy is needed.

En bloc extended total thymectomy and EPP was performed with extended incision of a posterolateral thoracotomy. During the surgery, meticulous attention was needed to prevent droplet metastasis. In order to prevent tumor cell spillage, black silk 3-0 sutures were used immediately when tearing of the parietal pleura occurred during extrapleural dissection. In two cases (patients 5 and 7) requiring resection of the innominate vein, a median sternotomy followed the posterolateral thoracotomy. Performing a posterolateral thoracotomy followed by a median sternotomy will lessen the risk for pleural droplet metastasis in the opposite thoracic cavity. If EPP is performed after opening the opposite pleura, gravity may enhance the possibility of droplet metastasis on the opposite side.

Selection criteria for the EPP must be considered. First, the patient's functional status has to be good enough to tolerate the pneumonectomy. Second, there should be no metastatic disease in the opposite thorax or the extrathoracic cavity. In this series, the PET-CT was used to rule out distant metastases. Third, complete or nearly complete resection should be expected when performing an EPP. Otherwise, a palliative approach should be considered in inoperable cases. The CT has been used to examine tumor invasion of neighboring organs, such as the heart, great vessels, and chest wall. Usually, the innominate vein and the superior vena cava can be safely resected. For patient 7, preparations were made for cardiopulmonary bypass because of concerns about aortic arch invasion. Fortunately, there was no need for aortic arch replacement; aortic invasion was not considered an absolute contraindication to the procedure. Forth, if a limited resection can achieve a complete resection, in individual pleural and pulmonary lesions, the EPP should be reserved for where it is most effective.

In this series, the outcome of extrapleural pneumonectomy was favorable with low morbidity and no mortality. However, the follow-up duration for this study was comparatively short for assessment of late recurrence and long term survival. In the future, multicenter trials are needed to establish standard treatment using a multimodality therapy including surgical procedures.

## Conclusions

En bloc extended total thymectomy and extrapleural pneumonectomy was safe and effective in selected patients with Masaoka stage IVA thymomas and can be expected to achieve complete local control. Although the treatment strategy has yet to be standardized, complete resection with appropriate systemic therapy should improve survival in an otherwise fatal disease.

## Abbreviations

EPP: extrapleural pneumonectomy; PET-CT: positron emission tomography - computed tomography; PTFE: polytetrafluoroethylene; CT: computed tomography; SVC: superior vena cava VATS: video assisted thoracoscopic; CAP: cyclophosphamide, doxorubicin and cisplatin; DOD: dead of disease; NED: no evidence of disease; S: surgery; RT: radiotherapy; CT (only shown in Table 1): chemotherapy; CAV: cyclophosphamide, doxorubicin, vincristine; VIP: etoposide, ifosfamide,

cisplatin.

## Competing interests

The authors declare that they have no competing interests.

## Authors' contributions

JK conceived of the study, and participated in its design and coordination and helped to draft and revise the manuscript for important intellectual content. HCY had full access to all of the data and takes responsibility for the integrity and accuracy of the data analysis and wrote all sections of the manuscript. YSY participated in the study design and helped to collect of data. HKK supervised the manuscript drafting. YSC advised and interpreted of data. KK participated in critical revision of the manuscript. YMS participated in critical revision of the manuscript. JH carried out the review of pathologic slides. All authors read and approved the final manuscript.
